# Production of functional, stable, unmutated recombinant human papillomavirus E6 oncoprotein: implications for HPV-tumor diagnosis and therapy

**DOI:** 10.1186/s12967-016-0978-6

**Published:** 2016-07-28

**Authors:** Elena Illiano, Olivia Costantina Demurtas, Silvia Massa, Paola Di Bonito, Valerio Consalvi, Roberta Chiaraluce, Carlo Zanotto, Carlo De Giuli Morghen, Antonia Radaelli, Aldo Venuti, Rosella Franconi

**Affiliations:** 1 Department of Pharmacological and Biomolecular Sciences, University of Milan, Via Balzaretti 9, 20133 Milan, Italy; 2 Laboratory of Biomedical Technologies (SSPT-TECS-TEB), Department for Sustainability, Division of Health Protection Technologies, Italian National Agency for New Technologies, Energy and the Environment (ENEA), ‘Casaccia’ Research Centre, Via Anguillarese 301, 00123 Rome, Italy; 3 Laboratory of Biotechnology (SSPT-BIOAG-BIOTEC), Department for Sustainability, Division Biotechnology and Agroindustry, Italian National Agency for New Technologies, Energy and the Environment (ENEA), ‘Casaccia’ Research Centre, Via Anguillarese 301, 00123 Rome, Italy; 4 Department of Infectious Diseases, Istituto Superiore Sanità, Viale Regina Elena 299, 00185 Rome, Italy; 5 ‘A. Rossi Fanelli’ Department of Biochemical Sciences, University of Rome ‘La Sapienza’, P.le Aldo Moro 5, 00185 Rome, Italy; 6 Department of Medical Biotechnologies and Translational Medicine, University of Milan, Via Vanvitelli 32, 20129 Milan, Italy; 7 Catholic University ‘Our Lady of Good Counsel’, Tirana, Albania; 8 Cellular and Molecular Pharmacology Section, CNR Institute of Neurosciences, University of Milan, 20129 Milan, Italy; 9 HPV-UNIT, Ridait Department, Regina Elena National Cancer Institute, Via E. Chianesi 53, 00144 Rome, Italy

**Keywords:** HPV, E6 oncoprotein, Biomarker, Vaccines, Diagnostic

## Abstract

**Background:**

High-risk human papillomaviruses (HR-HPVs) types 16 and 18 are the main etiological agents of cervical cancer, with more than 550,000 new cases each year worldwide. HPVs are also associated with other ano-genital and head-and-neck tumors. The HR-HPV E6 and E7 oncoproteins are responsible for onset and maintenance of the cell transformation state, and they represent appropriate targets for development of diagnostic and therapeutic tools.

**Methods:**

The unmutated E6 gene from HPV16 and HPV18 and from low-risk HPV11 was cloned in a prokaryotic expression vector for expression of the Histidine-tagged E6 protein (His_6_-E6), according to a novel procedure. The structural properties were determined using circular dichroism and fluorescence spectroscopy. His_6_-E6 oncoprotein immunogenicity was assessed in a mouse model, and its functionality was determined using in vitro GST pull-down and protein degradation assays.

**Results:**

The His_6_-tagged E6 proteins from HPV16, HPV18, and HPV11 E6 genes, without any further modification in the amino-acid sequence, were produced in bacteria as soluble and stable molecules. Structural analyses of HPV16 His_6_-E6 suggests that it maintains correct folding and conformational properties. C57BL/6 mice immunized with HPV16 His_6_-E6 developed significant humoral immune responses. The E6 proteins from HPV16, HPV18, and HPV11 were purified according to a new procedure, and investigated for protein–protein interactions. HR-HPV His_6_-E6 bound p53, the PDZ1 motif from MAGI-1 proteins, the human discs large tumor suppressor, and the human ubiquitin ligase E6-associated protein, thus suggesting that it is biologically active. The purified HR-HPV E6 proteins also targeted the MAGI-3 and p53 proteins for degradation.

**Conclusions:**

This new procedure generates a stable, unmutated HPV16 E6 protein, which maintains the E6 properties in in vitro binding assays. This will be useful for basic studies, and for development of diagnostic kits and immunotherapies in preclinical mouse models of HPV-related tumorigenesis.

## Background

New human papillomavirus (HPV) types are continuously being described, and 174 have been completely characterized. Among these, 47 can infect the ano-genital area [1]. While high-risk (HR)-HPVs are commonly associated with cancer, low-risk (LR)-HPVs have mostly been identified in condyloma acuminatum [2], with HPV6 and HPV11 responsible for 90 % of all genital warts.

It is believed that all cervical cancers are caused by HPV infections, and that HPV16 and HPV18 are responsible for about 70 % of all cases [3]. HPV16 and HPV18 have also been shown to cause almost half the vaginal, vulvar, and penile cancers, while about 85 % of anal cancers are also caused by HPV16 [4]. HPVs, and HPV16 in particular, are associated with some head and neck squamous-cell carcinomas, and they are an independent risk factor for oropharyngeal cancers [5].

The incidence of HPV-associated oropharyngeal cancer has increased over the past 20 years, especially among men, and it has been estimated that HPVs will cause more oropharyngeal than cervical cancer in the United States by 2020 [6].

Currently, routine screening is foreseen only for cervical cancer, where viral DNA detection (usually by PCR-based tests) has been used by different countries for cervical tumor prevention programs. However, although these tests are highly sensitive for the detection of HPVs [7, 8], they are not indicative of possible evolution of pre-cancerous lesions to tumors. The current challenge is therefore to develop tests that can better distinguish self-resolving HPV infections from those that might progress to pre-cancer and to cancer.

Recently, HPV16 E6 serology was identified as a promising pre-diagnostic marker for HPV-driven cancers [9], as HPV16 E6 seropositivity has been found more than 10 years before diagnosis of oropharyngeal cancers [10]. It is also important to note that seropositivity is relatively common before diagnosis of anal cancer, although it is rare for other HPV-related ano-genital tumors [11].

E6 is a potent oncogene of HR-HPVs, and its role in progression to malignancy has been, and continues to be, explored [12]. The E6 oncoprotein of HPVs targets numerous cell pathways to promote viral DNA replication. It forms a complex with human E3-ubiquitin ligase E6-associated protein (E6AP), which can in turn target the p53 tumor-suppressor protein, leading to its ubiquitin-mediated degradation. In particular, E6 from HR-HPVs can block apoptosis, activate telomerase, disrupt cell adhesion, polarity and epithelial differentiation, alter transcription and G-protein signaling, and reduce immune recognition of HPV-infected cells [13, 14].

The HR-HPV E6 proteins are characterized by a PDZ binding motif (PSD95/DLG/ZO-1; a structural domain of 80–90 amino-acids) at the carboxy (C)-terminus (e.g., RTRRETQL for HPV16 E6). Proteins that contain multiple PDZ domains are frequently expressed in regions of cell-to-cell contact and alterations to these intercellular junctions can destroy tissue organization and favor dysplastic events. Through degradation of human discs large tumor suppressor (hDLG), the HR-HPV E6 proteins can also alter cell growth and polarity in response to cell contact [15]. Other E6 PDZ-domain-containing targets include the MAGI proteins, which are members of the membrane-associated guanylate kinase homolog (MAGUK) family of scaffold molecules that are involved in regulation of tight-junction assembly [16]. This function is significant for E6 oncogenic activity, as deficiency in cell polarization is a marker of tumor progression [17].

The E6 protein has about 150 amino-acids, and it is characterized by two conserved zinc-finger-like internal sequences (i.e., Cx_2_C-x_29_-Cx_2_C). These are joined by an inter-domain linker of 36 amino-acids, and are flanked by short N-terminal and C-terminal domains of variable lengths [18]. Endogenous E6 is expressed at very low levels in HPV-containing cells, and it is subject to nuclear import and export processes, which is consistent with its targeting of proteins located in either the nucleus [19] or the cytoplasm [20].

Due to difficulties in the production of recombinant full-length E6 protein in its native and soluble forms, there is a lack of clear information about its structure. One problem is associated with its high cysteine content (e.g., 14 cysteines in HPV16 E6). Substitutions of the nonconserved cysteines in E6 with serines did not affect its activity towards in vitro and in vivo p53 degradation, and only marginally helped to make it more soluble [12]. E6 fused to the C-terminus of carrier proteins, such as maltose-binding protein (MBP-E6) or glutathione-S-transferase (GST-E6), were overexpressed in *Escherichia coli* in a soluble form, thus allowing these fusion proteins to be purified by single-step affinity procedures [21, 22]. However, proteolytic removal of the carrier proteins (i.e., MBP, GST) led to rapid precipitation of the E6 protein [23].

The E6 protein, as either unfused or His-tagged, is mainly produced as inclusion bodies [24], but when it is fused to the C-terminus of MBP, it appears in the form of soluble, high-molecular-weight aggregates [25] that can spontaneously assemble into large organized ribbon structures [26]. To date, the preparation of the concentrated and soluble HPV16 E6 protein has required addition of a peptide corresponding to the cellular acidic leucine (L)-rich (LxxLL) motif of E6AP, substitution of nonconserved cysteines, and mutation of the dimerization surface in its N-terminal domain [27]. These conditions resulted in the crystallization of HPV16 E6 with the LxxLL peptides of E6AP [28]. Also, the structure of the E6/E6AP/p53 complex that is required for HPV-mediated degradation of p53 was solved recently using a mutated full-length E6 protein (named as HPV16 E6 4C/4S), the LxxLL motif of E6AP, and the core domain of p53 [29].

In the present study, we developed a procedure for production of the HPV16 His_6_-E6 protein in its wild-type but soluble form and at high yields. The structural properties of this novel His_6_-E6 protein were examined using UV, circular dichroism (CD), and fluorescence spectroscopy, with its aggregation in solution examined using 90° light scattering. Binding investigations with p53, PDZ1 (from MAGI-1), hDLG and E6AP using GST pull-down assays showed that this native His_6_-E6 protein retains its biological activity, which was also confirmed using in vitro degradation assays. Large amounts of the His_6_-E6 protein were obtained by modulation of several chemicophysical parameters and solvent conditions (e.g., temperature, oxidation–reduction conditions, buffer pH, detergents), which were also applied to the His_6_-E6 protein from HPV18 and HPV11. Furthermore, this novel His_6_-E6 protein was able to induce a stronger humoral immune response in immunized C57BL/6 mice than the His_6_-E6 protein prepared in its denatured form.

This study thus provides a method to produce a soluble, stable and functional E6 oncoprotein that might represent a novel tool for HPV diagnosis and therapy. Moreover, it opens up the possibility to obtain further information about the structure and functions of E6.

## Methods

### Bacterial strains and recombinant DNA techniques

The *E. coli* strains XL1 Blue, M15[pREP4], BL21(DE3), and JM109 were grown in Luria–Bertani broth (LB; Sigma-Aldrich Italia, Milan, Italy) or on LB agar plates, in the presence of 50 mg/L kanamycin or 100 mg/L ampicillin. *Escherichia coli* competent cells were transformed using standard methods. The plasmid DNA isolated from selected clones was purified using plasmid purification kits (Qiagen, Hilden, Germany) and analyzed using restriction enzymes (New England Biolabs Ltd, Ontario, Canada) and DNA sequencing.

### Construction of expression plasmids

The E6 genes from HR-HPV16, HR-HPV18, and LR-HPV11 were cloned in the pQE30 *E. coli* expression vector (Qiagen) to allow expression of the 6× His-tagged recombinant proteins. The encoding DNA fragments were obtained from the pGEX plasmid using *BamH* I and *Not* I, and were ligated to pQE30 that had previously been cut with the same restriction endonucleases.

### Construction of co-expression plasmids

The chaperone plasmid set from Clontech (Takara Bio Company, Mountain View, USA) was used. The kit consists of five different plasmids, with each one designed to express multiple molecular chaperones to enable optimal protein expression and folding. In particular, chaperone A was designed to express the products of the *dnaK*-*dnaJ*-*grpE groES*-*groEL* genes, chaperone B for the *groES*-*groEL* genes, chaperone C for the *dnaK*-*dnaJ*-*grpE* genes, chaperone D for the *groES*-*groEL*-*tig* genes, and chaperone E for the *tig* gene (Table 1). The preparation of a system to co-express the target HPV16 His_6_-E6 protein and one of the chaperones was carried out according to the manufacturer specifications.

**Table 1:** Different combinations of molecular chaperones and *E. coli* strains tested for HPV His_6_-E6 expression

*E. coli* strain	Combination^a^ according to chaperone^b^ (plasmid)				
	A (pG-KJE8)	B (pGro7)	C (pKJE7)	D (pG-Tf2)	E (pTf16)
M15 [pREP4]	–	++	++++	–	++
BL21 (DE3)	–	++	+	–	+
JM109	+	+++	+	–	+++++

± Qualitative estimation of yield

^a^All combinations included the pQE30 HPV16 His_6_-E6 plasmid

^b^Chaperone **A** carried the *dnaK*-*dnaJ*-*grpE groES*-*groEL* genes, **B** the *groES*-*groEL* genes, **C** the *dnaK*-*dnaJ*-*grpE* genes, **D** the *groES*-*groEL*-*tig* genes, **E** the *tig* gene

The different *E. coli* strains were transformed with the pQE30-HPV16 His_6_-E6 expression plasmid along with one of each of these different chaperones. Each transformant that contained both plasmids (one expressing the HPV16 His_6_-E6 gene, and one expressing one of the chaperones) was induced. Purification was performed by affinity chromatography using Ni-nitrilotriacetic acid agarose resin (Ni-NTA; Qiagen). The total amount of His_6_-E6 was determined by sodium dodecyl sulfate-poly acrylamide gel electrophoresis (SDS-PAGE) and immunoblotting.

### Set-up of optimal parameters for expression and purification of the native His_6_-E6 protein

The His_6_-E6 protein was initially expressed for 3 h at 37 °C in the *E. coli* XL1 Blue strain in the absence of any chaperone. The induction and purification were performed by changing different chemico-physical parameters (Table 2, Protocols A–E), and by analyzing the products obtained using SDS-PAGE and immunoblotting. In particular, to improve the oxidation–reduction conditions and to avoid oxidation of free cysteines, 100 µM dithiothreitol (DTT) was added to the purification buffers. Buffers with lower pH than in standard protocols and with higher imidazole concentrations were also used, to move away from the HR-HPV16 E6 isoelectric point (pI = 9.24) and to better protect against contaminants [30].

**Table 2:** Chemical and physical parameters analyzed during the purification of the HPV16 His_6_-E6 protein under native conditions

Protocol	Temperature (°C)	Dithiothreitol (µM)	pH	Imidazole (mM)			Detergent^a^
				Lysis buffer	Wash buffer	Elution buffer	
A	Room temperature	–	8	10	20	250	–
B	4	100	8	10	25	250	–
C	4	100	8	20	50	250	–
D	4	100	7.5	20	70	300	–
E	4	100	7.5	20	70	300	0.02 % lauryl-β-D-maltoside 0.02 % Tween-20 0.02 % glycine 0.02 % betaine 0.1 M arginine
F	4	100	7.5	20	70	300	0.02 % betaine

^a^The detergents listed were tested individually

After optimization of the protocol, the His_6_-E6 protein was expressed overnight at 28 °C in *E. coli* JM109 with pTf16 (chaperone E, which expressed the trigger factor molecule; TF), which allowed high protein yields and facilitated its handling. The overnight culture was 100-fold diluted in fresh LB with 20 mg/L chloramphenicol and 100 mg/L ampicillin, and incubated at 37 °C until an OD_600_ of 0.6–0.7 was reached. Protein expression was induced with 1 mM isopropyl-β-d-thiogalactopyranoside (IPTG; Qiagen), and the cells were grown at 28 °C for 16 h before harvesting by centrifugation at 4000×*g* for 20 min at 4 °C. The pellet harvested from a 5-L bacterial culture was resuspended in lysis buffer (50 mM NaH_2_PO_4_, 200 mM NaCl, 20 mM imidazole, 100 µM DTT, pH 7.5) containing EDTA-free protease inhibitors (complete protease inhibitor cocktail tablets; Roche, Basel, Switzerland) at the concentration recommended by the manufacturer. After adding 1 mg/mL lysozyme and 1 % Triton X-100, the cells were incubated for 1 h at 4 °C, and then sonicated on ice at a 10-Hz output (3× for 1 min) in an ultrasonic disintegrator (Soniprep 150, MSE, UK). Clarification was performed by centrifugation at 15,000×*g* for 45 min at 4 °C, and the supernatant was incubated for 16 h with 1 mL Ni-NTA that had previously been equilibrated in lysis buffer. After washing the Ni-NTA several times with 50 mM NaH_2_PO_4_, 200 mM NaCl, 70 mM imidazole, 100 µM DTT, pH 7.5, to reach a final OD_280_ of 0.01, the protein was eluted from the Ni-NTA using 50 mM NaH_2_PO_4_, 200 mM NaCl, 300 mM imidazole, 100 µM DTT, pH 7.5. The different fractions were run on 15 % SDS-PAGE.

To improve the solubility of the purified His_6_-E6 oncoprotein, different detergents were added to the elution fractions and to the dialysis buffer at low concentrations, including 0.02 % lauryl-β-D-maltoside, 0.02 % Tween-20, 0.02 % glycine, 0.02 % betaine, and 0.1 M arginine, with the aim being to mask the hydrophobic surface groups of the purified His_6_-E6 protein. After addition of each detergent to different aliquots of the same preparation of the purified His_6_-E6 protein, the preparations were analyzed before and after dialysis, as well as after high-speed centrifugation. The fractions enriched in the recombinant His_6_-E6 protein were pooled, dialyzed against Ca^2+^-free and Mg^2+^-free phosphate-buffered saline (PBS^–^) containing 0.02 % betaine, 100 µM DTT, pH 7.5, and concentrated using 10-kDa cut-off centriprep centrifugation (Amicon, Merck Millipore, Darmstadt, Germany). The concentration of the purified His_6_-E6 protein was determined by absorbance at 280 nm, using the molar extinction coefficient of His_6_-E6 (i.e., 21,275 M^−1^ cm^−1^), calculated according to Gill and von Hippel [31]. Purified proteins were quantified in Coomassie-stained SDS-PAGE by comparison with 0.25, 0.5 and 1 μg of BSA run in the same gel. The purified His_6_-E6 protein was stored at 4 °C until use.

To evaluate the purity of the protein samples, they were denatured by boiling for 3 min in 2× SDS sample buffer (100 mM Tris–HCl, pH 6.8, 200 mM DTT, 4 % SDS, 20 % glycerol, 0.02 % bromophenol blue), separated using 15 % SDS-PAGE, and stained using Coomassie blue.

### Immunoblotting

The purified proteins in 2× SDS sample buffer were separated by SDS-PAGE and transferred onto PVDF membranes (Millipore, Darmstadt, Germany). The primary antibody was anti-E6 mouse polyclonal serum produced according to a previously published protocol [32] and utilized at a 1:1000 dilution [33]. The secondary antibody was a horseradish peroxidase (HRP)-conjugated sheep anti-mouse IgG antibody (1:10,000 dilution; GE Healthcare, Buckinghamshire, UK). Proteins were visualized using the chemiluminescent HRP substrate (Millipore, Darmstadt, Germany).

### UV, circular dichroism and fluorescence emission spectroscopy

The UV–visible spectra were recorded using a double-beam spectrometer (Lambda 16; Perkin-Elmer Life Sciences) that was equipped with a thermal controller (Peltier) set at 20 °C.

The CD spectra were recorded at 20 °C using a spectropolarimeter (Jasco J-720) equipped with a thermal controller (Peltier). Far-UV-CD spectra (190–250 nm) were measured in a 0.1–0.02-cm-path-length quartz cuvette, and near-UV-CD spectra (250–310 nm) in a 1.0-cm-path-length quartz cuvette. The data are expressed as the mean residue ellipticity ([Θ]), assuming a molecular weight of 110 Da for each amino-acid residue.

The measurements of the intrinsic fluorescence emission and 90° light scattering were carried out at 20 °C in a spectrofluorimeter (LS50B; PerkinElmer Life Sciences, Waltham, USA) using a 1-cm-path-length quartz cuvette. The intrinsic fluorescence emission spectra were recorded from 300 to 450 nm (at 1-nm sampling intervals) with the excitation wavelength at 290 nm. In all of the experiments, the slit widths were set to 3 nm for excitation and 5 nm for emission. The 90° light scattering was recorded at the wavelength of 480 nm both for excitation and emission.

The spectra were accumulated four times. All of the values were corrected for solvent contributions (PBS^–^ containing 100 µM DTT, 0.02 % betaine). The analysis of the far-UV-CD spectra was performed using the Dicroprot program [34] and the Dicroprot server [35], and by comparing different methods.

### GST pull-down assay

In vitro GST pull-down assays were used to determine any interactions between the E6 protein and its cellular protein targets (i.e., p53, PDZ1 from MAGI-1, hDLG, E6AP), expressed in *E*. *coli* XL1 as GST-fusion proteins. Briefly, *E*. *coli* XL1 were grown overnight (16 h), 100-fold diluted in fresh LB with 100 mg/L ampicillin, and incubated at 37 °C until an OD_600_ of 0.6–0.8 was reached. The expression of the recombinant proteins was induced with 1 mM IPTG for 3 h at 37 °C. The proteins were then purified and immobilized on glutathione-agarose (Sigma-Aldrich). All of the immobilized GST-fusion proteins were run on SDS PAGE to evaluate the amount of each protein. Similar amounts (as evaluated by Coomassie stained gels) of the GST- fusion proteins GST-p53, GST-PDZ1, GST-hDLG, GST-E6AP and GST-alone (as negative control) were incubated with 50 ng of E6 proteins from HPV16, HPV18, and HPV11 for 2 h at 4 °C, to determine any binding interactions.

After removing the supernatant, the resin was washed five times with PBS^–^ containing 0.25 % Nonidet-P 40. The assays were carried out three times. The purified His_6_-E6 protein (20 ng) from HPV16, HPV18, and HPV11 was used as a positive control. The proteins were analyzed using SDS-PAGE and immunoblotting.

### In vitro transcription-translation and degradation assays

The DNA of pSP64-HPV16 E6, pSP64-p53-pro and pCDNA3-MAGI-3 plasmids was transcribed and translated in vitro in a rabbit reticulocyte lysate (Promega TNT System, Madison, Wisconsin, USA), following the manufacturer instructions, with radiolabeling with 0.6 mCi/mL ^35^[S]-cysteine (GE Healthcare). The translation efficiency was monitored by analyzing 1 µL aliquots of each protein using SDS-PAGE and PhosphorImager analysis (Fujifilm, Milano, Italy).

In the standard in vitro degradation assay [36], degradation was monitored by mixing the translated target proteins with 50 ng of the purified His_6_-E6 protein at a 3:1 ratio with an incubation at 25 °C. After 1 h and 2 h, 5-µL aliquots were removed from the reaction mixtures and analyzed. All volumes were equalized using the water-primed reticulocyte lysate TNT mix (Promega). The samples were added to 5× loading buffer (250 mM Tris–HCl, pH 6.8, 10 % SDS, 30 % glycerol, 5 % β-mercaptoethanol, 0.02 % bromophenol blue), boiled and analyzed using SDS-PAGE and autoradiography. The degradation experiments were carried out three times, and the relative quantification was performed with a PhosphorImager by measuring the signal intensities of protein bands.

### Animal immunization with the HPV16 His_6_-E6 protein

Female 6–8-week-old C57BL/6 mice were used (Charles River; Como, Italy). Groups of five mice were injected subcutaneously on days 0, 7, 15, and 21. Each mouse was inoculated with a 100 µL volume containing 20 µg purified HPV16 His_6_-E6 protein, as produced under native or denaturing conditions, plus Freund incomplete or MF59 adjuvant. Saline solution was used for the control group. The mice were maintained under specific pathogen-free conditions, following the institutional guidelines.

### ELISA

Serum samples were collected from the immunized mice 1 week after the fourth boost. Sera from the mice in the same group were pooled and analyzed for E6-specific antibodies using ELISA, as described previously [37]. Briefly, 96-well maxisorp microtiter plates (Nunc, Naperville, IL, USA) were coated with the His6-E6 protein (200 ng/well, in PBS^–^ or carbonate buffered saline). The sera were diluted 1:100 in 2 % milk PBS, and the binding was detected using a HRP-conjugated sheep anti-mouse IgG antibody (1:10,000; GE Healthcare, Buckinghamshire, UK). The immunocomplexes were revealed by addition of the 2,2 azino-di-3-ethylbenz-thiazoline sulfonate substrate (Sigma-Aldrich), and the absorbance was read at 450 nm using a microtiter plate reader (Bio-Rad Laboratories, Hercules, CA, USA).

### Statistical analyses

Statistical analyses were performed using one-way ANOVA parametric tests and Bonferroni analysis of variance, using the GraphPad Prism 5 software. The significance was set as p < 0.05 (*), p < 0.01 (**), and p < 0.001 (***).

## Results

### Glycine, arginine and betaine detergents reduce degradation of the HPV16 E6 protein produced under native conditions

**Fig. 1:**
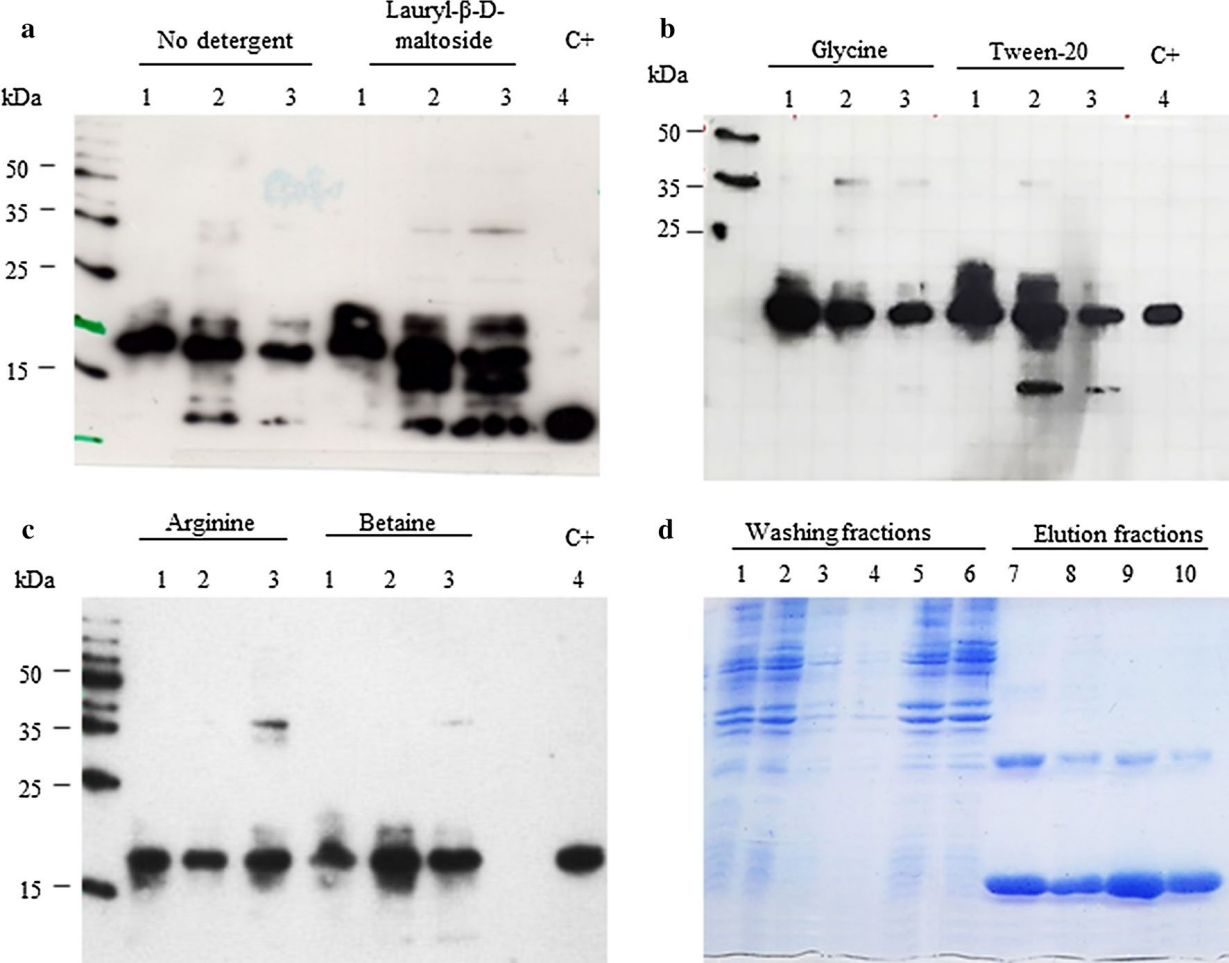
Effects of detergents on HPV16 His_6_-E6 solubility. Representative immunoblots of the soluble HPV16 His_6_-E6 protein before dialysis (*lanes 1*), after dialysis (*lanes 2*), and after high-speed centrifugation (*lanes 3*), in the absence of detergent (**a**) and with addition of lauryl-β-D-maltoside (**a**), glycine (**b**), Tween-20 (**b**), arginine (**c**) and betaine (**c**). C+ , HPV16 His_6_-E6 protein purified under denaturing conditions. (**d**) Representative SDS-PAGE of the HPV16 His_6_-E6 protein purified through the optimized protocol

With the aim to develop a standard protocol for expression and purification of the His_6_-E6 protein from different HPVs, the HPV16 His_6_-E6 protein was initially purified from inclusion bodies under denaturing conditions using immobilized metal-affinity chromatography. After dialysis, this His_6_-E6 protein showed a strong tendency to form insoluble aggregates, which generated a visible precipitate (data not shown). However, after lowering the temperature and adding DTT as a reducing agent, the His_6_-E6 protein was maintained in solution for a few hours. Other chemical and physical parameters were then varied (Table 2, Protocols A–E) to improve the characteristics of this His_6_-E6 protein. In the presence of lauryl-β-D-maltoside or Tween-20, the His_6_-E6 protein tended to be degraded, particularly soon after the dialysis and high-speed centrifugation, while the addition of glycine significantly reduced this degradation after dialysis and centrifugation (Fig. 1a, b). In particular, Fig. 1c shows that when arginine or betaine were included before dialysis (Fig. 1c, lanes 1), after dialysis (Fig. 1c, lanes 2), and after the high-speed centrifugation (Fig. 1c, lanes 3), only a single band of the expected molecular weight was detected by a HPV16 E6-specific serum [32, 33], thus demonstrating the absence of protein degradation. Figure 1d shows the SDS-PAGE of the HPV16 His_6_-E6 protein purified through this optimized protocol, and Table 2 indicates the different systems that were tested before the novel final purification procedure (Protocol F).

### HPV16 His_6_-E6 protein expression and purification are enhanced in the *E. coli* JM109 strain and with TF chaperone co-expression

To further enhance the His_6_-E6 expression levels, different *E. coli* strains were transformed with the HPV16 His_6_-E6 gene and different chaperone plasmids, for chaperone co-expressed. The results show that after induction with chaperones A to C, His_6_-E6 expression was always low with the *E. coli* BL21 strain, and improved with chaperone C and the *E. coli* M15 strain. However, the best results were obtained again using the TF chaperone with the *E. coli* JM109 strain (Table 1, chaperone E). This most favorable combination improved the His_6_-E6 protein yield, which resulted in the production of about 1.5 mg of HPV16 His_6_-E6 protein from 24 g of pelleted *E. coli* JM109 from 5 L of culture.

The induction of His_6_-E6 in *E. coli* JM109 was then analyzed by immunoblotting.

**Fig. 2:**
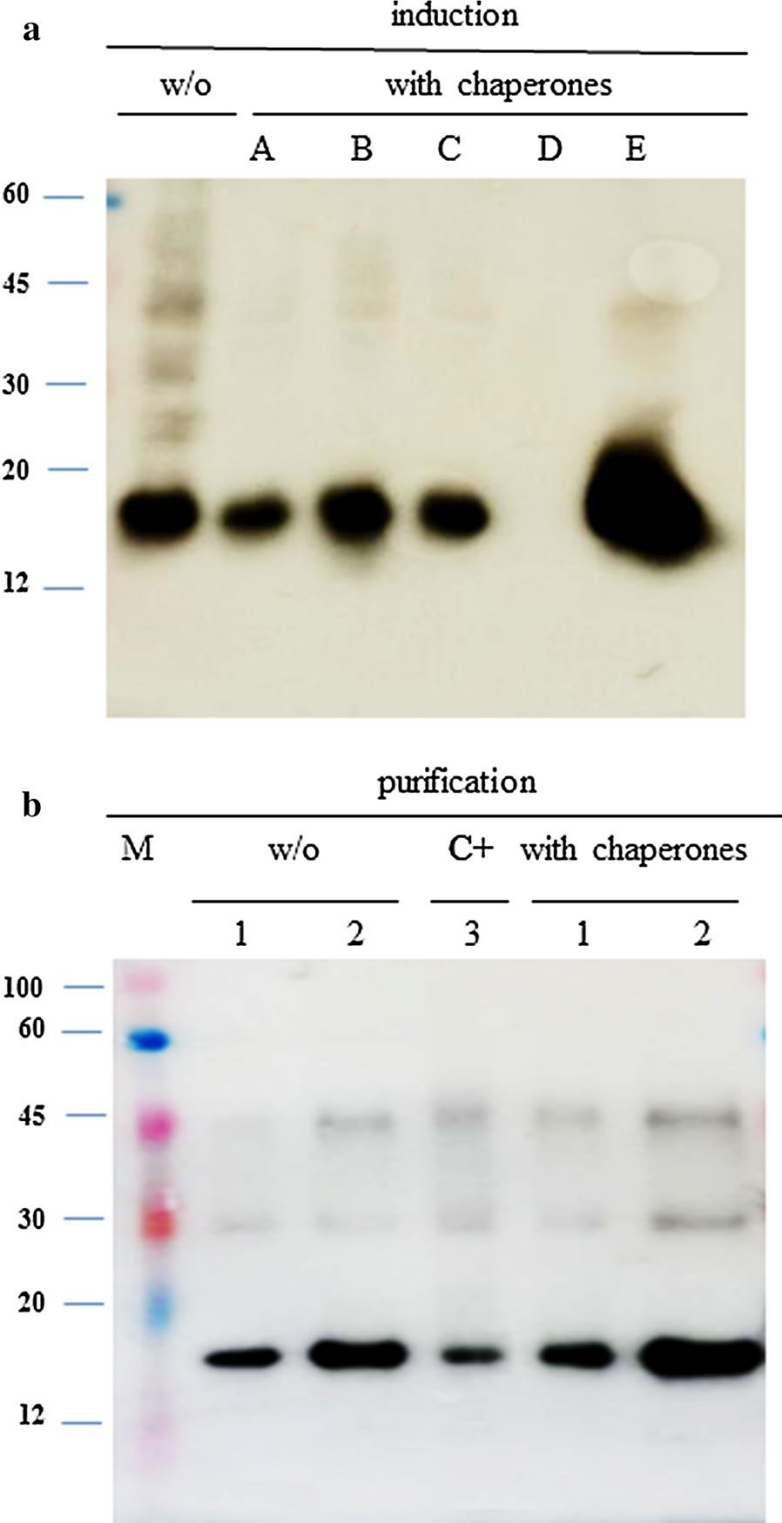
Effects of different chaperones on HPV His_6_-E6 expression levels. Representative immunoblots of induction and purification of HPV16 His_6_-E6 produced in *E. coli* JM109. **a** Bacterial transformation with the pQE30-HPV16 His_6_-E6 expression plasmid alone (w/o) or with the different chaperone systems (chaperones A–E, see Table 2). Total proteins were extracted by re-suspension in SDS-loading buffer of *E. coli* cell cultures, according to their OD_600_. **b** Purification of the HPV16 His_6_-E6 protein without chaperones (w/o) and with the trigger factor chaperone (with chaperones; chaperone E, see Table 2). *M* ColorBurst, Sigma, *lanes 1* 1 µL, *lanes 2* 3 µL, *lane 3* C^+^, 20 ng of purified His_6_-E6 protein positive control

After induction with chaperones A to C (Fig. 2a–c), His_6_-E6 expression was comparable to that obtained in the absence of chaperones (Fig. 2a, w/o) whereas it was very low, or even absent, when using chaperone D (Fig. 2a, d). Conversely, the use of the TF chaperone greatly enhanced the His_6_-E6 production (Fig. 2a, e). When the His_6_-E6 protein produced in *E. coli* JM109 with this TF chaperone were purified (Fig. 2b), there was more His_6_-E6 in the presence (Fig. 2b, with chaperones) than in the absence (Fig. 2b, w/o) of the chaperone. The conditions shown in Fig. 2b included 1 and 3 µL of these protein samples (lanes 1 and 2, respectively), with the His_6_-E6 protein purified under denaturing conditions used as the positive control (Fig. 2b, c^+^). Faint bands, corresponding to the molecular weights of E6 dimer and trimer, are also detectable.

### Spectroscopic analysis reveals the structure of the HPV16 His_6_-E6 protein purified under native conditions

**Fig. 3:**
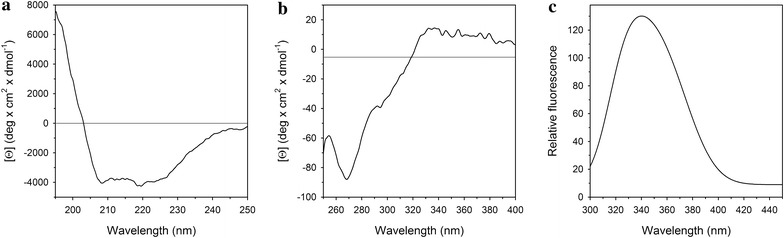
Spectral properties of the HPV16 His_6_-E6 protein. **a** Far-UV-CD spectrum. **b** Near-UV-CD spectrum. **c** Intrinsic fluorescence emission spectrum (290 nm excitation). All spectra were recorded at 20 °C in PBS^−^ containing 100 µM DTT and 0.02 % betaine

The HPV16 His_6_-E6 structure in solution was evaluated using CD and fluorescence emission spectroscopy (Fig. 3). The far-UV-CD spectrum was characterized by low ellipticity, a zero intercept at 203 nm, and similar contributions at 222 and 208 nm (Fig. 3a). These far-UV-CD spectral features are typical of proteins that have few elements of periodic secondary structure. The analysis of the far-UV-CD spectra identified approximately 12 % α-helices, 35 % β-sheets and 53 % random coils. The near-UV-CD spectrum of His_6_-E6 (Fig. 3b) showed broad ellipticity bands that were poorly resolved, at around 266 nm due to the presence of phenylalanine residues, and at around 292 nm, as a region that can generally be ascribed to tryptophan residues. In addition, a very large but poorly defined band of positive ellipticity was observed above 300 nm. The intrinsic fluorescence spectra of His_6_-E6 was recorded at 290 nm excitation wavelength, and this revealed maximum emission at 338 nm, a wavelength that is lower than that of tryptophan in water (around 350 nm) (Fig. 3c). No particle aggregation was detectable by 90° light scattering at 480 nm (data not shown).

### HPV His_6_-E6 proteins purified under native conditions are stable

**Fig. 4:**
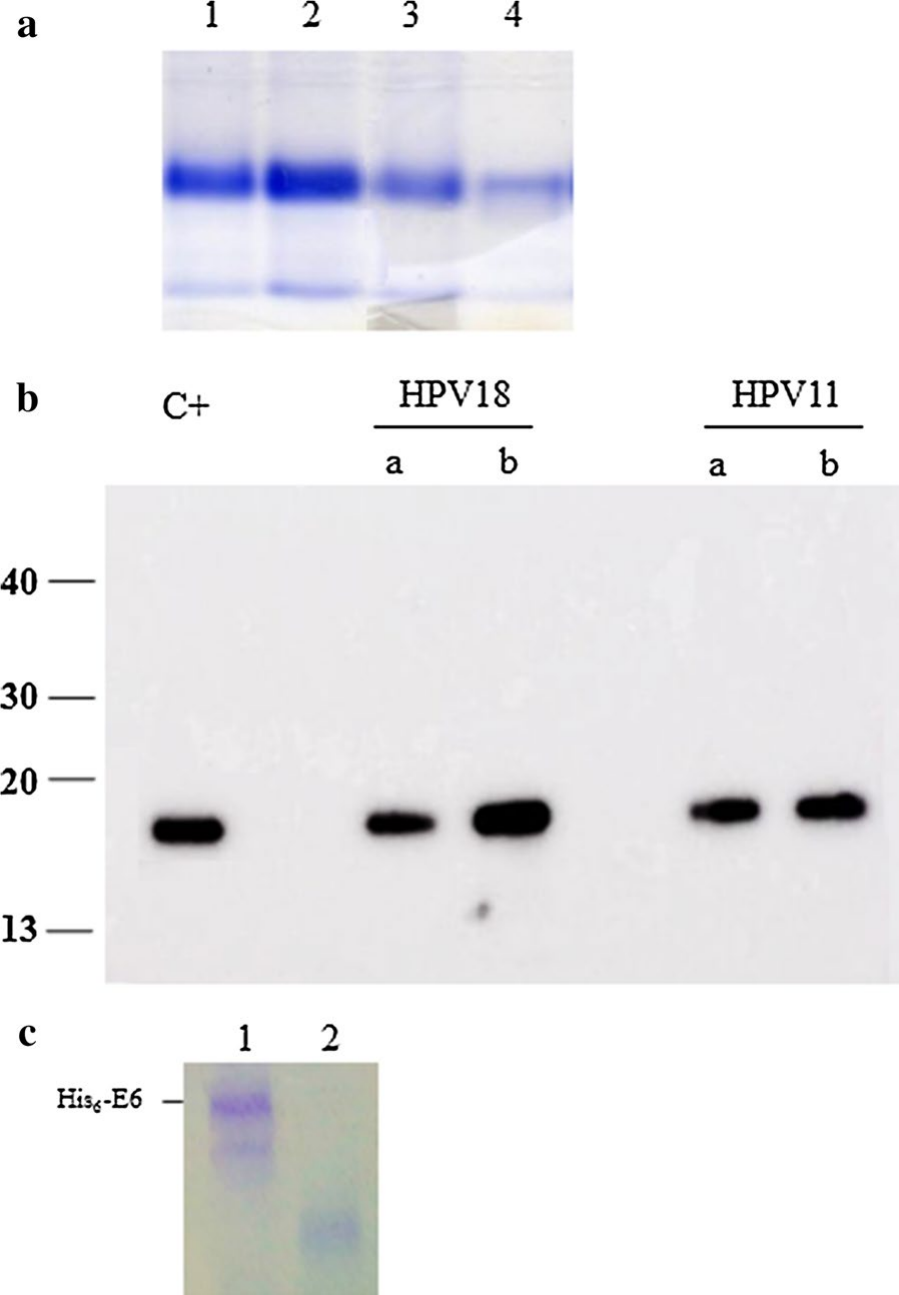
Purification of the HPV His_6_-E6 proteins under native conditions. The optimized protocol for HPV16 His_6_-E6 protein production was applied to the E6 protein from the different HPVs. **a** Coomassie blue staining of representative SDS-PAGE of purified E6 protein from HPV11 (*lane 2*), HPV16 (*lane 3*) and HPV18 (*lane 4*) after one chromatographic step. *Lane 1*, HPV16 His_6_-E6 protein produced under denaturing conditions, as control. **b** Representative immunoblot of the HPV18 and HPV11 His_6_-E6 proteins purified under native conditions before (**a**) and after (**b**) dialysis and concentration. C+ , concentrated HPV16 His_6_-E6 protein, as control. **c** Coomassie blue staining of representative SDS-PAGE of HPV16 His_6_-E6 protein stored for more than 2 years at 4 °C (*lane 1*) and −20 °C (*lane 2*)

Once the protocol was set up for HPV16 His_6_-E6, the same protocol was used for the purification of the His_6_-E6 protein from HR-HPV18 and LR-HPV11. Only one chromatographic step was necessary to purify the His_6_-E6 proteins from HPV16, HPV18, and HPV11 to homogeneity (i.e., ~90 %), as shown by Coomassie blue staining following SDS-PAGE (Fig. 4a, lanes 2, 3, 4, respectively). Denatured HPV16 His_6_-E6 was used as the control (Fig. 4a, lane 1). No protein degradation was observed in the immunoblots of the His_6_-E6 proteins purified under native conditions from HPV18 and HPV11 both before (Fig. 4b, lanes a) and after (Fig. 4b, lanes b) the dialysis and concentration steps. The concentrated HPV16 His_6_-E6 protein was used as the control here (Fig. 4b, c^+^).

### HPV His_6_-E6 proteins purified under native conditions show biological activity

**Fig. 5:**
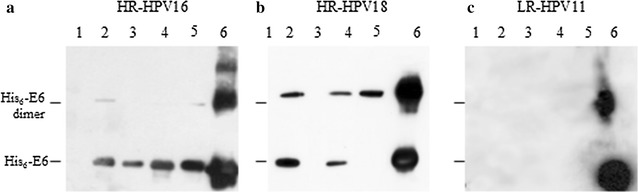
Physical interactions between purified HPV His_6_-E6 proteins and their cellular targets. Representative immunoblots from GST pull-down assays of the His_6_-E6 proteins from HR-HPV16 (**a**), HR-HPV18 (**b**), and LR-HPV11 (**c**) for the GST-fusion proteins for DLG (*lanes 2*), E6AP (*lanes 3*), p53 (*lanes 4*), and PDZ of MAGI-1 (*lanes 5*). *Lanes 1* GST-protein as negative controls, *lanes 6* purified His_6_-E6 proteins, as positive controls

To determine whether the HPV His_6_-E6 proteins from HR-HPV16, HR-HPV18 and LR-HPV11 retain biological activity through this new purification protocol, these proteins were purified under native conditions and analyzed in vitro using GST pull-down assays for the GST-hDLG, GST-E6AP, GST-p53, and GST-PDZ of MAGI-1 fusion proteins purified from *E. coli* on glutathione agarose (Fig. 5, lanes 2, 3, 4, 5, respectively). The binding activities toward these cellular proteins showed that HPV16 His_6_-E6 bound its most important cellular targets mainly as a monomer (Fig. 5a), whereas HPV18 His_6_-E6 bound hDLG, p53, and PDZ1 both as a monomer and a dimer (Fig. 5b, lanes 2, 4, 5), but did not bind E6AP (Fig. 5b, lane 3). Conversely, there were no interactions between LR-HPV11 His_6_-E6 and the tested targets (Fig. 5c). The purified His_6_-E6 proteins that were used as the positive controls showed dimeric bands for all of the HPVs (Fig. 5, lanes 6), whereas there were no bands with free GST protein, used as the negative control (Fig. 5, lanes 1).

### HPV His_6_-E6 proteins purified under native conditions can degrade their targets

**Fig. 6:**
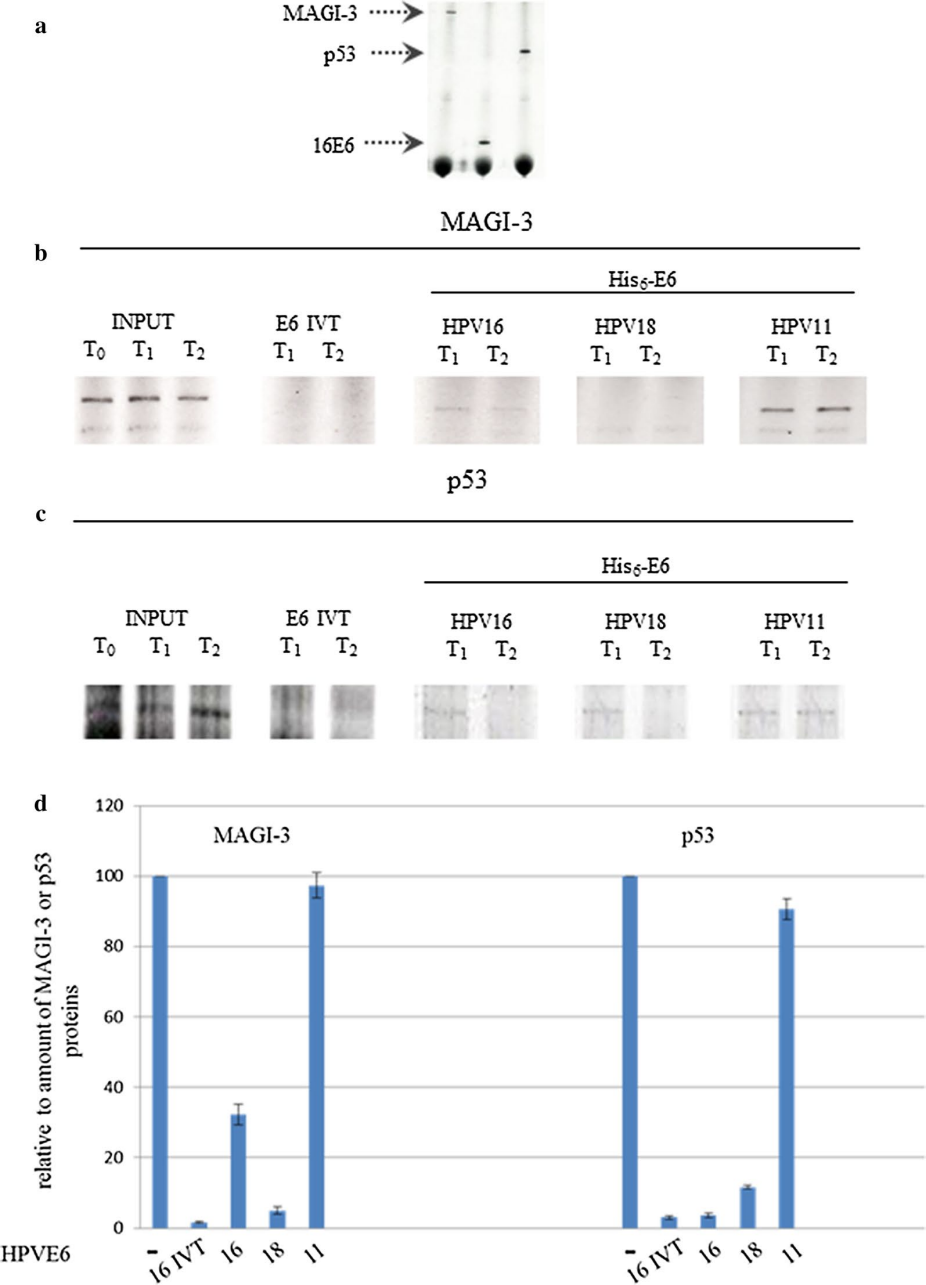
In vitro degradation of p53 and MAGI-3 by HPV His_6_-E6 proteins. HPV16 E6, MAGI-3, and p53 proteins were in vitro translated in rabbit reticulocyte lysates. **a** Representative autoradiography for these ^35^[S]-cysteine-radiolabeled proteins following SDS-PAGE for the protein translation efficiencies (as indicated). **b**, **c** Representative SDS-PAGE for degradation of MAGI-3 (**b**) and p53 (**c**) following their mixing with purified HPV His_6_-E6 proteins (as indicated). The reaction mixtures were sampled after 1 h (T_1_) and 2 h (T_2_) of incubation. *INPUT* without purified His_6_-E6 protein, *E6 IVT* in vitro transcribed E6 protein from HPV16, as positive control (**d**). Quantification of MAGI-3 and p53 degradation was achieved by measuring the signal intensities of protein bands with a PhosphorImager. Data represent the mean of three independent experiments ± standard deviations. Values are presented relative to the amount of MAGI-3 or p53 in the presence of water-primed reticulocyte lysate, after 2-h incubation

The in vitro translational efficiencies of MAGI-3, HPV16 E6, and p53 before their use in the degradation assays are shown in Fig. 6a, lanes 1, 2, 3, respectively. The HPV His_6_-E6 purified proteins were then mixed with either MAGI-3 (Fig. 6b) or p53 (Fig. 6c), to determine whether they degraded these target proteins. Aliquots of each reaction mixture after incubations of 1 h (T1) and 2 h (T2) were analyzed by SDS-PAGE. When MAGI-3 was combined with HPV16 or HPV18 His_6_-E6, a very weak band for MAGI-3 was seen at T1 that had almost disappeared at T2. In contrast, this band was seen at both T1 and T2 for HPV11 His_6_-E6 (Fig. 6b). Similar results were observed with p53, where a very weak p53 band was seen at T1 for all three HPV His_6_-E6 proteins, but only for HPV11 His_6_-E6 at T2. As controls, MAGI-3 and p53 proteins were incubated in the absence of His_6_-E6 and the in vitro translated HPV16 E6 protein alone was used in the incubations (Fig. 6). Reported data are the result of three independent experiments (Fig. 6d).

### His_6_-E6 protein in its native form induces a better humoral immune response than in the denatured state

**Fig. 7:**
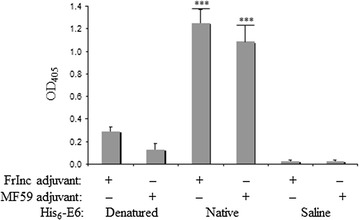
Anti-E6 humoral responses in mice. Mice were inoculated with 20 µg purified HPV16 His_6_-E6 protein (100 µL) produced under denaturing or native conditions in Freund incomplete (FrInc) or MF59 adjuvants, and with the control of the adjuvants alone (as indicated). Sera were collected 1 week after last boost immunization, pooled and analyzed by ELISA for the levels of E6-specific antibodies. ***p < 0.001 vs. denatured protein/control inoculation

The immunogenicity of the HPV16 His_6_-E6 protein was tested in vivo in a mouse model. As shown in Fig. 7, the mice were immunized with denatured or native purified His_6_-E6 with either Freund incomplete or MF59 adjuvant, with the control mice injected with PBS^–^ with the adjuvants. All of the mice received four subcutaneous inoculations, and a week after the last inoculation they were sacrificed and analyzed for the presence of antibodies against His_6_-E6. The mice immunized with native His_6_-E6 showed significantly higher titers of specific antibodies compared to those immunized with His_6_-E6 produced under denaturing conditions (Fig. 7; p < 0.001) and to the control mice (Fig. 7; p < 0.001). There were no significant differences seen between the use of the Freund incomplete or MF59 adjuvants.

## Discussion

HR-HPVs are responsible for more than 550,000 new cases of cervical cancer each year, and have also been implicated in vaginal, vulvar, and penile cancers. A strong link also exists between infection by HR-HPVs and the development of anal and oropharyngeal tumors [4].

Although the HPV E6 protein has been studied for several decades, the crystallographic structure was only recently solved for the HPV16 E6 protein with a modified amino-acid sequence and stabilized by fusion with the cellular LxxLL motive of E6AP [28]. A key to successful analysis of the structure and function of a recombinant protein is its efficient expression and purification, which is also an important goal for the development of therapeutic drugs. For this purpose, *E. coli* is the most commonly used host for the expression of recombinant proteins, although these proteins are often produced in inclusion bodies where they can assume an insoluble, non-native conformation.

Production of the full-length E6 protein as inclusion bodies is relatively easy, and our preliminary expression and purification also showed that this E6 protein was present in visible aggregates, with the soluble and native conformations of E6 difficult to obtain. To overcome this problem, the temperature was the first parameter considered here, as it represents a fundamental condition through purification steps, and as different proteins have various degrees of thermal stability. This was kept at 4 °C, to guarantee the solubility of the E6 protein. To prevent His_6_-E6 denaturation or degradation, the pH was also varied in the different protocols. This was defined on the basis of keeping it closer to the intracellular pH and far from the E6 isoelectric point, as proteins show minimum solubility at their isoelectric point. In this way, after affinity chromatography with Ni-NTA, the His_6_-E6 oncoprotein was recovered in a highly purified form (~90 %), which was aided by the addition of increasing concentrations of imidazole to the purification buffers. Also, 100 µM DTT was added to the purification buffer as a reducing agent, to prevent the formation of disulfide bridges between the several cysteine residues of the E6 amino-acid sequence. Low levels of different detergents were also tested, to improve the His_6_-E6 solubility after purification, where glycine, arginine and betaine also prevented protein degradation. This appeared to occur through hindering the formation of the hydrophobic interactions that lead to His_6_-E6 aggregation and precipitation. Betaine was finally adopted as the detergent of choice to improve the protein solubility, as it also did not interfere with the spectroscopic analyses [38].

By combining these parameters in this novel purification protocol, visible aggregation was no longer present, and the His_6_-E6 protein remained soluble and stable for more than 2 years at 4 °C (Fig. 4c).

This protocol was also applied to the purification of the HPV18 and HPV11 His_6_-E6 proteins, although lower yields were obtained for the LR-HPV E6 protein, which might be explained by its lower isoelectric point (pI = 8.44) compared to HR-HPV E6 (pI = 9.24).

To enhance the His_6_-E6 protein expression levels, different *E. coli* strains were also tested and combined with different molecular chaperones. It is known that chaperones can assist in the covalent folding/unfolding and assembly/disassembly of other macromolecular structures, and several chaperone systems can carry out a multitude of functions, all of which are aimed at insuring correct folding of the target proteins [39]. The highest HPV16 His_6_-E6 protein yield was obtained using the *E. coli* JM109 strain plus the TF chaperone molecule, which is crucial in the first step of protein synthesis and assists protein folding mainly by accelerating peptidyl-prolyl cis-trans isomerization [40]. Due to the relatively high proline content of the E6 protein (HPV16, HPV18, and HPV11 contain 7, 6, and 4 proline residues, respectively), the isomerization of peptide bonds with proline might be fundamental for its folding.

The HPV16 His_6_-E6 protein conformation was then analyzed by spectroscopy. This analysis revealed a low content of periodic secondary structure, which might be in agreement with the binding of E6 to many target proteins. The positive ellipticity band at >300 nm might be due to the effects of disulfides as chromophores, as previously described by Mulkerrin [41] and Hennecke [42]. The low degree of periodical secondary structure of the HPV16 His_6_-E6 protein was accompanied by its poorly defined tertiary arrangement, as determined by near-UV-CD, while the fluorescence spectrum suggested that the tryptophan residue was not exposed to the solvent. This might be explained according to bioinformatic studies on the HPV proteome (E6 and E7 oncoproteins in particular) where it emerged that these proteins can be traced back to a family of intrinsically disordered proteins [43]. Taken together, these analyses suggest that HPV16 His_6_-E6 retains correct folding and conformational properties.

HPV16 His_6_-E6 also appeared to be functional in terms of its activity. Different assay systems were used to confirm the functionality of the HPV His_6_-E6 proteins, including their interactions with E6AP, p53, and PDZ domains (i.e., DLG, MAGI-1), and their degradation of p53 and MAGI-3. When the His_6_-E6 oncoproteins were used in the GST pull-down assays, a second, higher molecular weight band was seen (in particular for HPV18 His_6_-E6), which corresponded to their dimeric forms. Considering the oxidation-prone behavior of the E6 protein, such dimers probably form via cysteine bonds, which suggests that higher concentrations of reducing agents in the storage buffer might help to keep these His_6_-E6 proteins in the monomeric form. It has also been reported that the HPV11 E6 protein can bind p53 and E6AP [44], but in the present study, no such interactions were observed between HPV11 His_6_-E6 and the targets included here.

To determine the immunogenicity of the native His_6_-E6 protein, mice were immunized using HPV16 His_6_-E6 combined with two alternative adjuvants, Freund incomplete or MF59. Only the His_6_-E6 produced under native conditions induced strong humoral responses, with no significant difference between the use of these two adjuvants. Thus, these data might indicate that the E6 with a non-conformational structure lost its immune-dominant antigenic sites, and suggest that the native E6 protein might be more useful as an immunogen.

In summary, the results from the present study thus indicate that: (i) the E6 protein produced in its native condition is protected from degradation by glycine, arginine, and betaine; (ii) His_6_-E6 protein expression levels and purification are enhanced in the *E. coli* JM109 strain and with TF chaperone co-expression; (iii) the spectroscopic analysis confirms the native structure of the purified HPV16 His_6_-E6 protein; (iv) the purified His_6_-E6 protein shows biological activity; (v) His_6_-E6 binds to the cellular target proteins that are responsible for E6 oncogenic activity, and it can promote in vitro degradation of the MAGI-3 and p53 cellular targets; and (vi) only the native His_6_-E6 protein induces good humoral immune responses.

## Conclusions

This novel protocol designed around a prokaryotic expression system allows biologically active His_6_-E6 proteins from HR-HPV16, HR-HPV18, and LR-HPV11 to be obtained at high yields in their native, soluble and stable forms. In particular, the purified HPV16 His_6_-E6 protein was stable for more than 2 years when stored at 4 °C, and was immunogenic in the mouse model. This antigen might also be useful to obtain monoclonal or polyclonal antibodies, which are still lacking as reagents for in vivo (e.g., ‘imaging’) or in vitro (e.g., immunoblotting, immunohistochemistry) diagnosis. This new procedure (that was set up through the present study) might also be useful to prepare the E6 protein for immunotherapy of HPV-related cancers, and to favor its industrial production for diagnostic tests (i.e., Luminex technologies).

With this aim, the maintenance of the native conformation of the E6 protein should improve the specificity, costs, precision, and reproducibility in the detection of anti-E6 antibodies in patient sera.

## Abbreviations

DLG 1: discs large
E6AP: E6-associated protein
HR/LR-HPVs: high/low-risk human papillomaviruses
MAGI-1: membrane-associated guanylate kinase with inverted domain structure 1
PDZ domain: PSD-95⁄ DLG⁄ ZO-1 domain
GST: glutathione S-transferase
IPTG: isopropyl thio-b-d-galactoside
MBP: maltose-binding protein
PBS^–^: Ca^2+^-free and Mg^2+^-free phosphate-buffered saline
